# A 3D in vitro model of the human breast duct: a method to unravel myoepithelial-luminal interactions in the progression of breast cancer

**DOI:** 10.1186/s13058-017-0843-4

**Published:** 2017-04-21

**Authors:** Edward P. Carter, James A. Gopsill, Jennifer. J. Gomm, J. Louise Jones, Richard P. Grose

**Affiliations:** 1 Centre for Tumour Biology, Barts Cancer Institute - a Cancer Research UK Centre of Excellence, Queen Mary University of London, London, EC1M 6BQ UK; 2 Department of Mechanical Engineering, University of Bristol, Bristol, BS8 1TR UK

## Abstract

**Background:**

3D modelling fulfils a critical role in research, allowing for complex cell behaviour and interactions to be studied in physiomimetic conditions. With tissue banks becoming established for a number of cancers, researchers now have access to primary patient cells, providing the perfect building blocks to recreate and interrogate intricate cellular systems in the laboratory. The ducts of the human breast are composed of an inner layer of luminal cells supported by an outer layer of myoepithelial cells. In early-stage ductal carcinoma in situ, cancerous luminal cells are confined to the ductal space by an intact myoepithelial layer. Understanding the relationship between myoepithelial and luminal cells in the development of cancer is critical for the development of new therapies and prognostic markers. This requires the generation of new models that allows for the manipulation of these two cell types in a physiological setting.

**Methods:**

Using access to the Breast Cancer Now Tissue Bank, we isolated pure populations of myoepithelial and luminal cells from human reduction mammoplasty specimens and placed them into 2D culture. These cells were infected with lentiviral particles encoding either fluorescent proteins, to facilitate cell tracking, or an inducible human epidermal growth factor receptor 2 (HER2) expression construct. Myoepithelial and luminal cells were then recombined in collagen gels, and the resulting cellular structures were analysed by confocal microscopy.

**Result﻿s:**

Myoepithelial and luminal cells isolated from reduction mammoplasty specimens can be grown separately in 2D culture and retain their differentiated state. When recombined in collagen gels, these cells reform into physiologically reflective bilayer structures. Inducible expression of HER2 in the luminal compartment, once the bilayer has formed, leads to robust luminal filling, recapitulating ductal carcinoma in situ, and can be blocked with anti-HER2 therapies.

**Conclusions:**

This model allows for the interaction between myoepithelial and luminal cells to be investigated in an in-vitro environment and paves the way to study early events in breast cancer development with the potential to act as a powerful drug discovery platform.

**Electronic supplementary material:**

The online version of this article (doi:10.1186/s13058-017-0843-4) contains supplementary material, which is available to authorized users.

## Background

The ducts of the human breast are composed primarily of two cellular elements in a bilayer structure: luminal epithelial cells, which form a polarised layer around the central ductal cavity, and myoepithelial cells that are positioned between the basement membrane and the luminal epithelial layer. These myoepithelial cells secrete extracellular matrix components required for the correct polarity of the luminal cells and also contract during lactation in order to propel milk through the ductal tree [1, 2].

An intriguing relationship between these two cell types is observed in ductal carcinoma in situ (DCIS). DCIS is characterised by a proliferation of neoplastic luminal cells into the luminal space of the breast duct, whereas the outer ring of myoepithelial cells remains intact. Accordingly, many have proposed that DCIS is a precursor to invasive breast cancer [3, 4]. However, as many as 50% of DCIS cases will not develop into invasive breast cancer [5, 6]. Combined with earlier detection of DCIS, there has been a rise in potential overdiagnosis of breast cancer and, as a consequence, potentially unnecessary treatment [7]. Novel prognostic markers are therefore needed to identify which cases of DCIS will progress to invasive cancer and which will remain benign. Putative markers are likely to reflect either a loss of myoepithelial integrity, which facilitates subsequent cancer cell invasion, or an alteration in the myoepithelial phenotype [8]. Moreover, deeper understanding of how the myoepithelial cells maintain the polarised luminal surface, as well as how the relationship between luminal and myoepithelial cells alters with tumour evolution, is key to understanding early mechanisms of cancerous transformation in the breast.

2D cell culture is the primary tool for cancer researchers because this technique provides a standardised high-throughput system whereby the characteristics of specific cell types can be dissected and compared with those of other researchers across the world. However, this is largely reliant on cancer cell lines that do not adequately recapitulate the complexity of the cancer environment [9]. 3D culture systems are more advantageous because they better reflect the environment in vivo and allow the impact of the extracellular matrix to be assessed. For instance, a number of breast epithelial cell lines, such as MCF-10A cells, are able to form spheroids of polarised epithelial cells with luminal centres, similar to breast duct morphology, when placed in extracellular matrix gels [10–12]. However, a caveat to these systems is that there is no true bilayer structure, thus preventing dissection of the myoepithelial-luminal cell relationship.

Tissue banks for cancer research are becoming increasingly accessible (e.g., http://breastcancertissuebank.org), providing researchers with access to normal as well as tumour-derived patient cells. These invaluable resources require new methodologies and models to maximise their potential benefit for research and patients. Using cells derived from normal human breast tissue, we have developed a novel 3D model of the human breast duct bilayer for research applications. We demonstrate that our model is more reflective of the human breast duct than current models and highlight its translational utility by adapting a lentiviral engineering approach to allow objective evaluation of early-stage breast cancer.

## Results

### Primary human myoepithelial and luminal cells maintain their characteristics in vitro

**Fig. 1:**
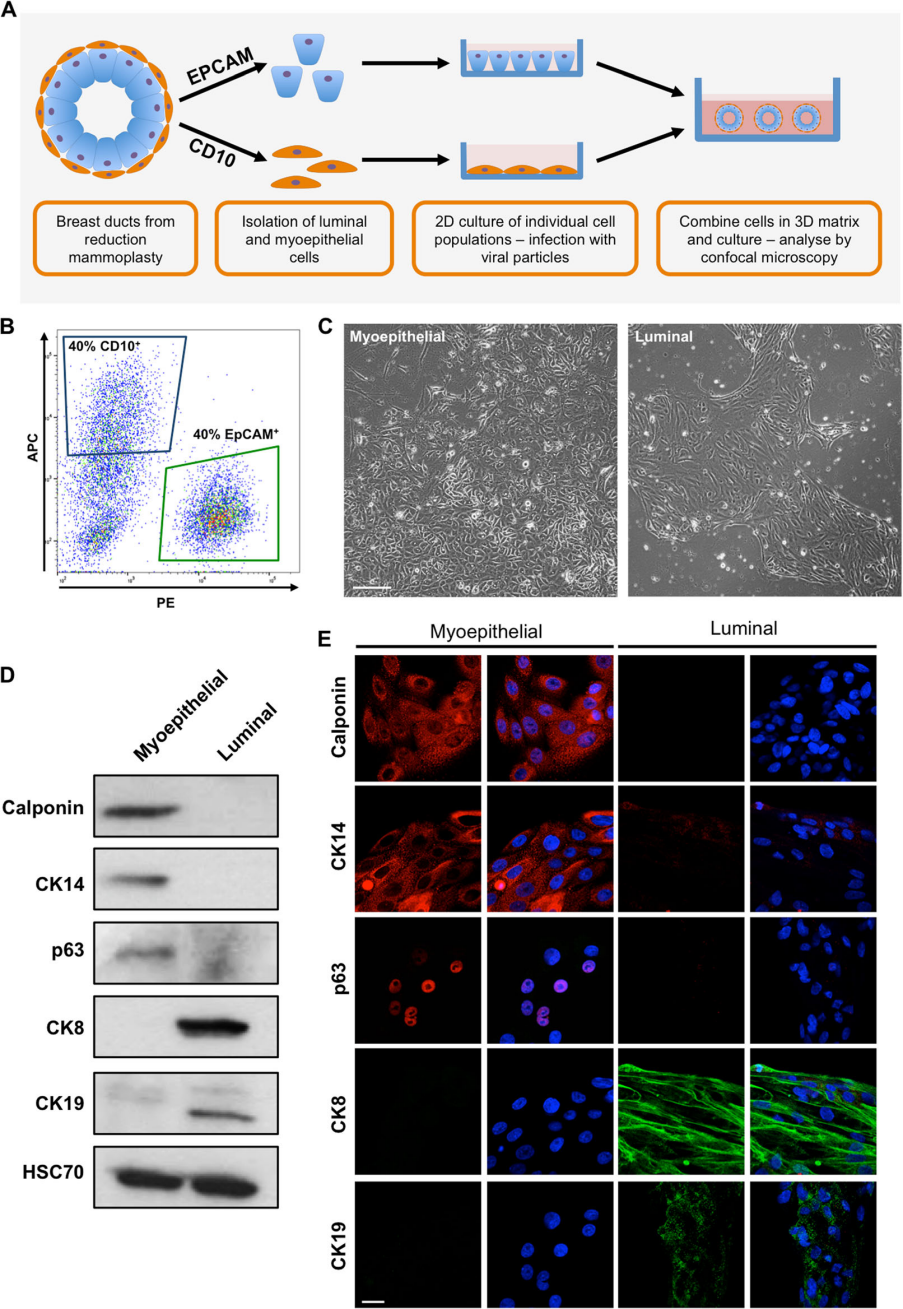
Isolated myoepithelial and luminal cells maintain their characteristics in vitro. **a** Schematic of proposed ductal model. **b** Representative fluorescence-activated cell sorting plots of reduction mammoplasty specimens separated by expression of CD10 (allophycocyanin fluorescence, *blue gate*) and epithelial cell adhesion molecule (EpCAM; phycoerythrin fluorescence, *green gate*). **c** Representative light micrographs of isolated myoepithelial and luminal cells grown in vitro for 10 days. Images taken at × 4 original magnification. Scale bar = 100 μm. **d** and **e** Western blot (**d**) and confocal (**e**) analysis of calponin, p63, cytokeratin (CK) 14, CK8 and CK19 expression in myoepithelial and luminal cells grown for 10 days in culture. Cell nuclei are labelled with 4′,6-diamidino-2-phenylindole (*blue*). Images and plots are representative of cells derived from at least three donors. Scale bar = 20 μm

To adequately investigate the relationship between myoepithelial and luminal cells, these two cell populations first need to be separated and cultured individually to allow for their genetic manipulation prior to rebuilding the duct in vitro (Fig. 1a). These two cell types were separated from ductal organoids isolated from patients who had undergone reduction mammoplasty, based on their expression of CD10 and epithelial cell adhesion molecule (EpCAM), for myoepithelial and luminal cells, respectively (Fig. 1b). Luminal and myoepithelial cells can be maintained and proliferate in specific culture medium and exhibit distinct morphologies (Fig. 1c). It has been suggested that the mouse mammary gland contains adult stem cells that can proliferate into both cell types of the ductal tree [13, 14]. We therefore assessed whether our cultures of purified myoepithelial and luminal cells maintained their differentiated phenotypes in culture. Following 10 days of 2D culture, myoepithelial cells expressed the myoepithelial markers calponin, cytokeratin (CK) 14 and p63 and showed no expression of the luminal markers CK8 and CK19 (Fig. 1d, e). The converse was found with cultured luminal cells. It was noted that a few luminal cells expressed low levels of CK14. The appearance of CK14-positive luminal cells has, however, been reported in the normal human breast duct [15]. We suspect this small CK14-positive population simply reflects the heterogeneity of the luminal cells extracted from our preparations. Whereas luminal cells could be maintained in culture for 3 weeks before becoming senescent, myoepithelial cells could be cultured for up to 8 weeks, and during this time, both cell types retained their characteristics. Overall, our analysis suggests that neither cell type transdifferentiates while in culture.

### Myoepithelial and luminal cells form physiologically reflective ductal structures in 3D collagen gels

**Fig. 2:**
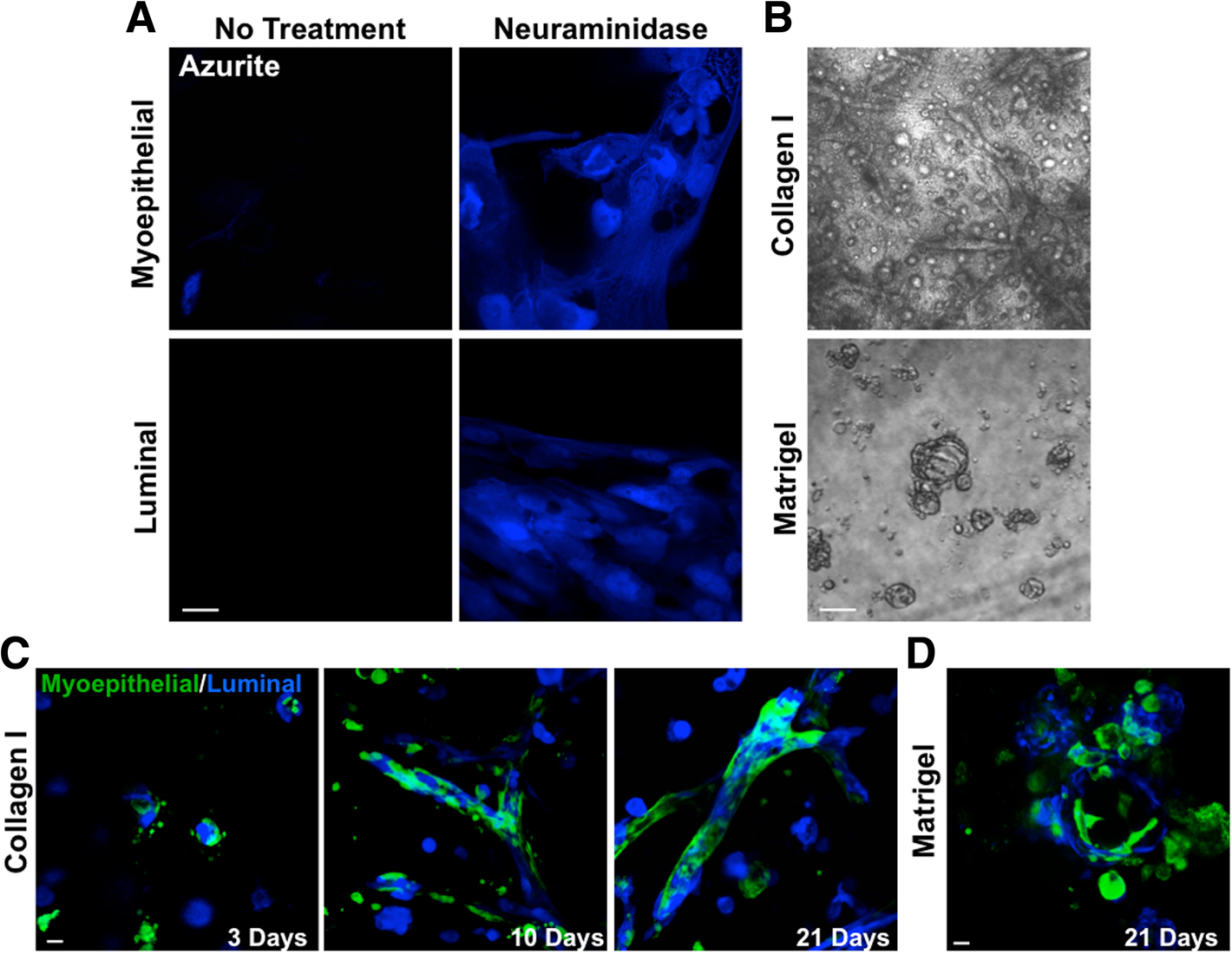
Myoepithelial and luminal cells are susceptible to lentiviral infection and re-form into spheroid and ductal structures in collagen-based gels. **a** Azurite blue expression in myoepithelial and luminal cells 48 h following lentiviral delivery with or without neuraminidase (20 mU/ml) pre-treatment. Scale bar = 20 μm. **b** Representative bright-field images of isolated myoepithelial and luminal cells grown together for 21 days in collagen- or Matrigel-based gels. Scale bar = 200 μm. **c** and **d** Representative confocal images of green fluorescent protein-labelled myoepithelial and azurite blue-labelled luminal cells grown in collagen-based (**c**) or Matrigel-based (**d**) gels. Images are representative of cells derived from at least three donors. Scale bar = 20 μm

To examine the fate of these two cell types in 3D gels, we introduced genetic constructs for fluorescent proteins into the cells prior to their recombination. Mixed cultures of breast epithelial cells have been shown to be particularly resistant to lentiviral infection [16]. Indeed, we observed the same resistance to lentiviral infection in our individual cultures of myoepithelial and luminal cells, with negligible expression of fluorescent proteins upon exposure to azurite lentiviral particles (Fig. 2a). Lentiviral infection was substantially increased by pre-treating the virus with neuraminidase prior to the application of particles to cells (Fig. 2a) [16]. To effectively manipulate transgene expression in these primary cells, it is important to ascertain the most efficient promoters that can be used. We examined the ability of several common promoters (*CMV*, *EF1α*, *CAG*, *PGK* and *UBC*) to drive green fluorescent protein (GFP) expression in both luminal and myoepithelial cells. While cytomegalovirus (CMV) was able to drive significant protein expression in both cell types, all other promoters were very weak (Additional file 1: Figure S1).

3D culture models use a variety of matrix substrates to facilitate the growth of cellular structures. Matrigel, a solubilised basement membrane preparation extracted from Engelbreth-Holm-Swarm mouse sarcoma cells, and rat-tail collagen are two of the most common matrices used for 3D culture. We therefore decided to test both types of matrices for ductal re-formation using isolated luminal and myoepithelial cells. Our fluorescence-activated cell sorting (FACS) analysis confirmed previous data indicating that myoepithelial and luminal cells exist at a 1:1 ratio in the normal human breast ducts (Fig. 1b) [17]. Cultured myoepithelial and luminal cells were added to 3D gels at this ratio to best mimic the physiological state. When placed as a mixture of single cells into collagen gels, spheroid and elongated ductal structures were formed by 10 days of culture, which became more complex by day 21. Matrigel-based gels, however, supported only spheroid formation (Fig. 2b).

Using GFP-labelled myoepithelial cells and azurite blue-labelled luminal cells in collagen-based gels, we observed that these two cell types began to coalesce by 3 days of culture. By 7 days, defined structures had formed, with myoepithelial cells forming an outer ring around an inner core of luminal cells, which became more complex by day 21 (Fig. 2c). Myoepithelial cells were observed to adopt an elongated morphology along the ducts, recapitulating their native state. Conversely, when placed in Matrigel, myoepithelial and luminal cells formed spheres comprised of cell types in no defined arrangement (Fig. 2d).

**Fig. 3:**
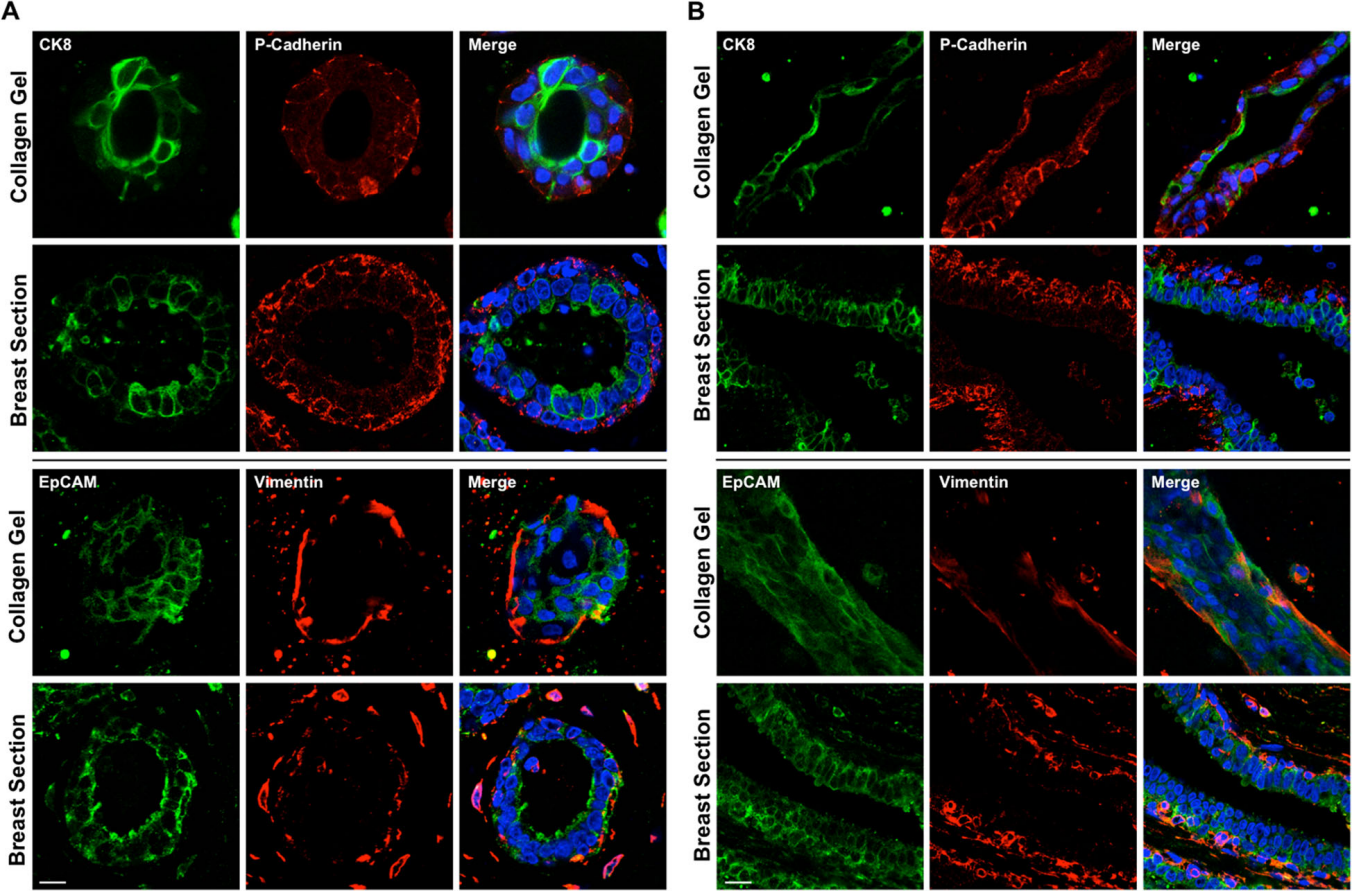
Spheroid and ductal structures formed in collagen gels recapitulate a physiological breast bilayer. Expression of cytokeratin (CK) 8, P-cadherin, epithelial cell adhesion molecule (EpCAM) and vimentin spheroid (**a**) and ductal (**b**) structures formed from myoepithelial and luminal cells grown in collagen for 21 days. Representative images from sections of normal human breast ducts (**a** and **b**) are also presented. Cell nuclei are labelled with 4′,6-diamidino-2-phenylindole (*blue*). Scale bar = 20 μm. Images are representative of structures formed from cells derived from at least three donors

We next determined the composition of the structures formed by examining the expression of myoepithelial and luminal markers and comparing these with normal human sections. Spheroids and ducts formed in collagen gels consisted of an inner ring of cells expressing luminal markers (CK8, EpCAM) surrounding a central luminal cavity. This inner layer was surrounded by an outer layer expressing markers of myoepithelial cells (P-cadherin, vimentin), consistent with the bilayer seen in normal human duct sections (Fig. 3). The cells comprising spheroids formed in Matrigel expressed both myoepithelial and luminal markers and lacked a bilayer configuration (Additional file 1: Figure S2).

### HER2 overexpression destabilises ductal bilayer

This model of the ductal bilayer allows for the manipulation of either cell type prior to recombination. We wished to examine whether this model could be used to study prominent drivers of breast cancer in a more physiological human context. Human epidermal growth factor receptor 2 (HER2) overexpression is found in approximately 30% of invasive breast cancers and is associated with aggressive disease progression [18]. HER2 is also overexpressed in many high-grade DCIS lesions. Although the HER2-targeted therapies, such as trastuzumab and lapatinib, are effective in the clinic [18, 19], many patients do not respond to treatment, and as many as 70% will develop resistant cancers [20]. Thus, there is a need to interrogate the biology of HER2-driven cancers to help overcome acquired resistance.

**Fig. 4:**
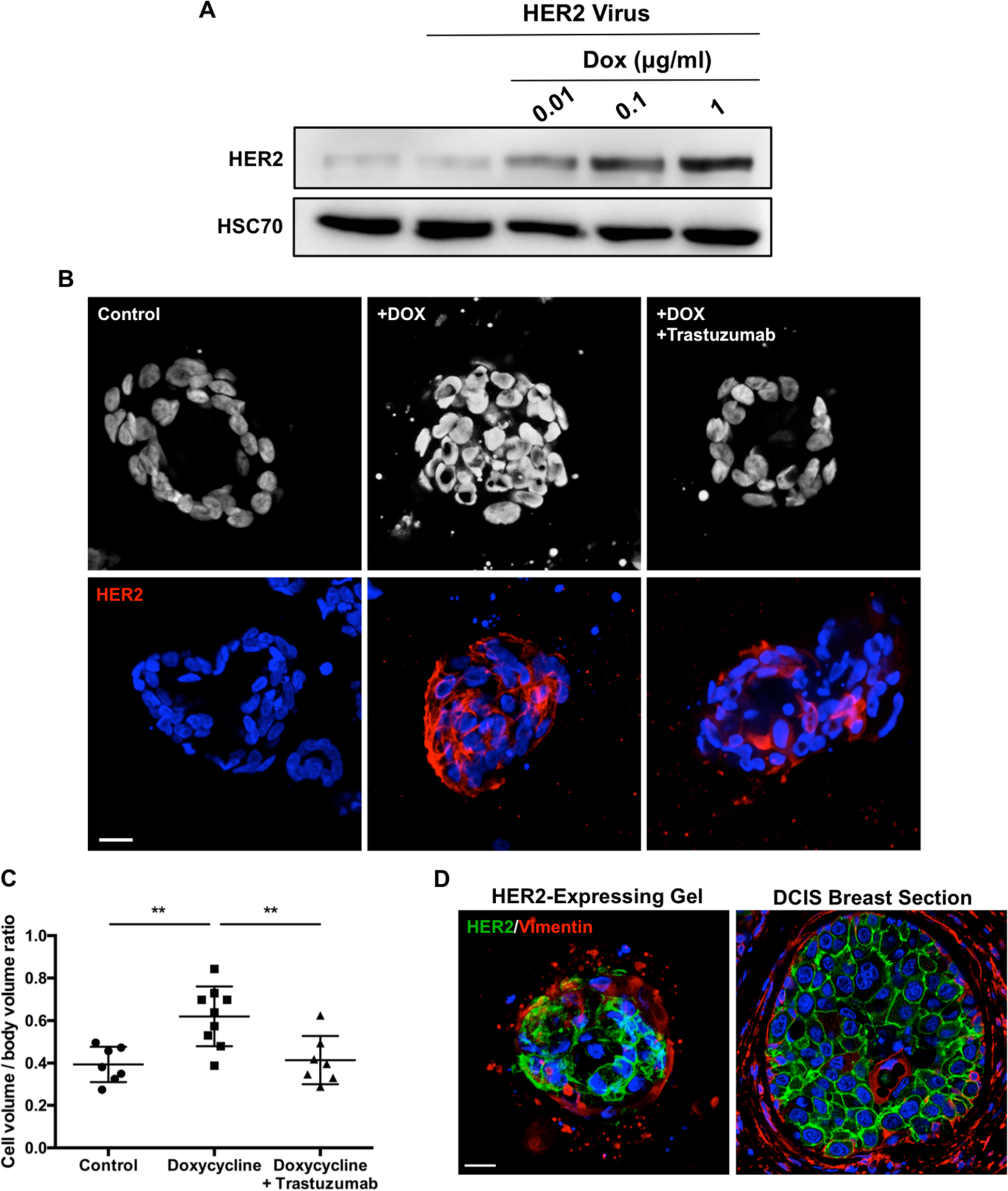
Overexpression of human epidermal growth factor receptor 2 (HER2) in the luminal compartment of a bilayer model results in destabilisation of the bilayer and luminal filling. **a** HER2 expression in luminal cells infected with inducible HER2 lentiviral particles and cultured in 0.01, 0.1 or 1 μg/ml doxycycline (DOX) for 48 h. **b** Representative z-sections of 4′,6-diamidino-2-phenylindole (DAPI)-labelled nuclei (*upper panels*) and HER2 expression (*lower panels*) of ductal spheroids following 14 days of culture plus 7 further days with (*middle panel*) or without (*left panel*) 1 μg/ml DOX treatment. Alternatively, DOX-treated cultures were also treated with 10 μg/ml trastuzumab for the final 7 days of culture (*right panel*). Scale bar = 20 μm. **c** Quantification of spheroids following treatment presented as ratio of total cell volume to total sphere volume. **d** HER2 and vimentin expression in representative HER2-expressing ductal spheroids and ductal carcinoma in situ breast sections. Cell nuclei are labelled with DAPI (*blue*). Scale bar = 20 μm. Data are presented as mean ± SD. Images are representative of structures formed from cells derived from at least three donors. ** *P* < 0.01

Fine control of gene expression is essential for its study in complex 3D models; stable overexpression of HER2 would allow for only its role in the formation of model bilayer to be studied, whereas inducible titred expression allows for change in gene expression to be assessed once a model bilayer has formed, mimicking the progression of breast cancer. We therefore constructed an inducible HER2 expression construct based on the pINDUCER system [21]. Expression of HER2 was induced readily and efficaciously in infected luminal cells upon exposure of the cells to doxycycline (Fig. 4a).

HER2-inducible luminal cells were combined with unaltered myoepithelial cells in collagen gels and cultured for 14 days, in which time ductal structures appear. Doxycycline, with or without trastuzumab, was then added to cultures for the remaining 7 days to induce HER2 expression. Control structures exhibited intact bilayer formation and a hollow luminal centre (Fig. 4b). Conversely, ducts treated with doxycycline for the final 7 days of culture exhibited significant luminal filling, with clear overexpression of HER2 in luminal cells compared with controls (Fig. 4b). Further, an intact myoepithelial layer was still evident in these cultures, as evidenced by an outer layer of vimentin-expressing cells (Fig. 4d). This expression pattern recapitulates that observed in HER2^+^ DCIS samples (Fig. 4d).

To objectively quantify luminal filling, we calculated the volume of cells within representative spheres and compared this with the calculated volume for the entire structure, and luminal filling would be signified by a significantly higher cell volume/total volume ratio than structures with complete lumen (Additional file 1: Figure S3). We observed a significant increase in cell/total volume ratio when this strategy was applied to cultures treated with doxycycline compared with untreated controls (Fig. 4c).

Strikingly, co-treatment with doxycycline and the HER2-targeted antibody trastuzumab prevented luminal filling, despite the overexpression of HER2 in the luminal compartment (Fig. 4b). This is further demonstrated by a significant reduction in cell/total volume of co-treated structures compared with doxycycline alone (Fig. 4c).

## Discussion

3D models are becoming increasingly commonplace as the technologies and associated imaging techniques become more accessible to researchers [22]. Complex models such as these require building blocks that best resemble the tissue they seek to mimic. Cancer cell lines in 3D environments are very useful for answering basic biological questions, but they are of little use when modelling the complex tumour architecture because many cell lines do not adequately reflect the tumour they purport to represent [9, 12]. Furthermore, they typically represent late-stage disease and not early disease and its interaction with components of the microenvironment. Primary cells derived from patient samples are the perfect resource for complex 3D models that seek to represent the normal and progressive tumour environment. We have demonstrated that normal myoepithelial and luminal cells can be isolated from reduction mammoplasty specimens and maintain their differentiated state in traditional 2D cultures. Recombined into a 3D environment, these cells reorganise into a native bilayer structure. This is a unique strength of our system compared with those that use common cell lines such as MCF10A cells, which can form spheroids with myoepithelial and luminal characteristics but lack a bilayer arrangement of cells.

The 3D environment in which the cellular components reside is a critical component of any model and modifies the resulting structure and behaviour. Our model highlights the importance of using an appropriate matrix where collagen-based gels promoted the re-formation of a bilayer from isolated myoepithelial and luminal cells, whereas Matrigel did not. Matrigel is rich in laminin, which is required for correct luminal orientation in the breast [23]. Myoepithelial cells are a major source of laminin in the breast [24]; therefore, in a Matrigel environment, there is no incentive for the two cell types to come together. However, in collagen, the luminal cells require myoepithelially expressed laminin, thus promoting their coalescence into co-units. It is intriguing that the cells in Matrigel cultures adopted characteristics of both myoepithelial and luminal cells. This would suggest that one of the many components of Matrigel is sufficient to drive the plasticity of these cells, which may have implications for the use of Matrigel in primary cell-based work.

Overexpression of HER2 in the luminal component of our bilayer model resulted in the destabilisation of the bilayer and filling of the luminal centre. The breakdown in epithelial orientation in response to overexpression of HER2 has been documented in cancer cell lines [25]. Strikingly, this loss of lumen could be blocked with HER2-targeted therapy. This proof of concept establishes the use of this model as a screen for novel therapies, not only for HER2-driven cancer but also potentially for any breast cancer subtype, given appropriate manipulation of the cellular components. Intriguingly, the outer myoepithelial layer of our model remained intact following HER2 overexpression in the luminal compartment, recapitulating the development of DCIS. Furthermore, this model can be modified to recapitulate the heterogeneity present in breast tumours. Heterogeneity within breast tumours accounts for variation in treatment response and the emergence of therapy-resistant tumours. Current animal- and cell-based models of breast cancer inherently model a homogeneous tumour and therefore are inadequate to study this crucial component of breast cancer [26]. The ability to objectively quantify luminal filling is a significant strength of this model because it demonstrates the reproducibility of our model between patient samples and confirms the appropriateness of this model for semi-high-throughput applications.

## Conclusions

A number of groups have developed complementary 3D models of the human breast duct from primary human cells, highlighting the need for such models in research [27–29]. The critical advantage of our model is the ability to isolate the individual cell types of the breast duct and modify them as desired before recombining them in 3D to re-create a physiologically reflective duct. This model has the potential to facilitate the dissection of cellular crosstalk in the breast and illuminate the relationship between myoepithelial and luminal cells in the progressive stages of breast cancer. Moreover, this offers a powerful and unique tool to understand the biology of DCIS and potential therapeutic approaches, which are essential to developing more tailored therapy for this poorly understood disease.

## Methods

### Cell isolation and culture

Ductal organoids from reduction mammoplasty specimens were obtained from the Breast Cancer Now Tissue Bank (REC 15/EE/0192). Ductal organoids were digested to a single-cell suspension through digestion in a 0.05% trypsin, 0.4 mg/ml DNase solution at 37 °C for 15 minutes as described previously [30]. Pure populations of myoepithelial and luminal cells were then isolated through either magnetic bead or FACS separation. Briefly, a single-cell suspension of cells derived from organoids was incubated at 4 °C for 20 minutes with a mouse anti-human CD10 antibody (catalogue number mca1556; Bio-Rad Laboratories, Oxford, UK) conjugated to sheep anti-mouse immunoglobulin G Dynabeads (Thermo Fisher Scientific, Paisley, UK) at a ratio of 2:1 (cells/beads) to label myoepithelial cells. Tagged cells were then pulled out through magnetic separation, and the remaining cells were incubated with Epithelial Enrich Dynabeads (Thermo Fisher Scientific) for 20 minutes at 4 °C to label luminal cells.

Alternatively, for isolation by FACS, single cells derived from organoids were resuspended at 20 × 10^6^ cells/ml and incubated with 0.25 μg/ml allophycocyanin (APC)-conjugated mouse anti-human CD10 (catalogue number 332777; BD Biosciences, Oxford, UK) and 0.06 μg/ml phycoerythrin (PE)-conjugated mouse anti-human EpCAM (catalogue number 347198; BD Biosciences) antibodies for 45 minutes at 4 °C. Cells were then incubated with 0.1 μg/ml 4′,6-diamidino-2-phenylindole (DAPI) to label dead cells prior to the separation of myoepithelial and luminal cells based on APC and PE fluorescence, respectively. FACS separation was performed on a BD FACSAria II cell sorter (BD Biosciences).

Isolated luminal cells were cultured in DMEM/F-12 medium (Sigma-Aldrich, Poole, UK) supplemented with 10% FBS, 0.5 μg/ml hydrocortisone (Sigma-Aldrich), 10 μg/ml apo-transferrin (Sigma-Aldrich), 5 μg/ml insulin (Sigma-Aldrich) and 10 ng/ml epidermal growth factor (EGF; Sigma-Aldrich). Isolated myoepithelial cells were cultured in HuMEC medium (Thermo Fisher Scientific) supplemented with 0.5 μg/ml hydrocortisone, 5 μg/ml insulin, 10 ng/ml EGF and 50 μg/ml bovine pituitary extract (Thermo Fisher Scientific).

### 3D ductal culture

Primary myoepithelial and luminal cells were combined in a 1:1 ratio and placed in collagen gels, consisting of 2 mg/ml collagen type I (Corning Life Sciences, Flintshire, UK), and 25 mM HEPES, prepared in luminal culture medium adjusted to neutral pH with NaOH. Gels were allowed to set at 37 °C before being overlaid with luminal culture medium. Culture media and indicated drug treatments were changed every 2–3 days. Alternatively, equal proportions of myoepithelial and luminal cells were placed on a pre-set bed of Matrigel Growth Factor Reduced (Corning Life Sciences) and maintained in luminal culture medium containing 5% Matrigel. Doxycycline was sourced from Sigma-Aldrich. Trastuzumab was a kind gift from Roche Pharma (Basel, Switzerland).

### Immunofluorescence

Cells cultured on coverslips were fixed in 10% neutral buffered formalin, permeabilised with 0.05% saponin and blocked with 5% bovine serum albumin (BSA) prior to incubation with primary antibody diluted in 5% BSA. Samples were incubated subsequently in a species-appropriate fluorescent secondary antibody before being mounted.

Collagen gels were first treated with 1 mg/ml collagenase (Sigma-Aldrich) for 10 minutes at 37 °C prior to fixation in 10% neutral buffered formalin. Gels were then permeabilised overnight with 1% Triton X-100 and blocked in 10% FBS/2% BSA. Gels were then incubated in primary antibodies for 48 h, followed by a 2-h incubation with species-appropriate fluorescent secondary antibody prior to mounting. Where indicated, gels were incubated in 1 μg/ml DAPI prior to mounting to label cell nuclei.

Paraffin-embedded sections of normal and DCIS breast tissue samples were obtained from the Breast Cancer Now Tissue Bank (REC 15/EE/0192). Sections were de-waxed, and antigen retrieval was performed though boiling sections with 10 mM sodium citrate buffer, pH 6.0. Sections were subsequently permeabilised in 0.1% Triton X-100 and blocked with 10% FBS/2% BSA. Sections were then incubated in primary antibodies as indicated above prior to secondary incubation in a species-appropriate fluorescent antibody. Prior to mounting, sections were incubated with 1 μg/ml DAPI to label cell nuclei.

Primary antibodies used were CK19 (catalogue number RB-9021; NeoMarkers/Thermo Fisher Scientific, Fremont, CA, USA), EpCAM (catalogue number MA5-12436; Thermo Fisher Scientific), p63 (catalogue number SC-8431; Santa Cruz Biotechnology, Dallas, TX, USA), vimentin (catalogue number HPA001762; Atlas Antibodies, Bromma, Sweden), P-cadherin (catalogue number 2198 s; Cell Signaling Technology, Danvers, MA, USA), HER2 (catalogue number 2165 s; Cell Signaling Technology), CK14 (catalogue number ab51054; Abcam, Cambridge, UK), CK8 (catalogue number C5301; Sigma-Aldrich), calponin (catalogue number ABT129; EMD Millipore, Watford, UK) and vimentin (catalogue number M0725; Dako, Carpinteria, CA, USA). Secondary antibodies used were Alexa Fluor 488 donkey anti-mouse (catalogue number A21202; Life Technologies) and Alexa Fluor 555 donkey anti-rabbit (catalogue number A31572; Life Technologies). Fluorescent images were acquired using a Carl Zeiss LSM710 confocal microscope (Carl Zeiss Microscopy, Thornwood, NY, USA).

### Immunoblotting

Cells lysates were prepared in 50 mM Tris-HCl, 150 mM NaCl, 1% Nonidet P40 buffer supplemented with protease (EMD Millipore) and phosphatase (EMD Millipore) inhibitor cocktails. Proteins were separated on a 10% SDS-PAGE gel, transferred onto a nitrocellulose membrane and blocked in 5% milk before being incubated in primary antibody diluted 1:1000 in 5% BSA. Membranes were then incubated with a species-appropriate HRP-conjugated secondary antibody (Dako) before bands were visualised using an enhanced chemiluminescence detection kit (GE Healthcare Life Sciences, Piscataway, NJ, USA). In addition to the primary antibodies listed above, the antibody HSC70 (catalogue number sc-7298; Santa Cruz Biotechnology) was used. HRP-linked secondary antibodies used were goat anti-mouse (catalogue number P0447; Dako) and goat anti-rabbit (catalogue number P0448; Dako).

### Lentiviral cloning and production

An inducible HER2 expression vector was constructed by subcloning the *ERBB2* open reading frame from pDONR223-*ERBB2* (23888; Addgene, Cambridge, MA, USA) into pINDUCER21 (46948; Addgene) using the Gateway LR Clonase kit (Thermo Fisher Scientific) following the manufacturer’s guidelines. Lentiviral particles were generated by co-transfecting HEK293T cells with the packaging plasmids pMD2.G (12259; Addgene) and pCMVR8.2 (12263; Addgene) and either pLV-GFP (36083; Addgene), pLV-Azurite (36086; Addgene) or pINDUCER21-*ERBB2* using FuGENE HD transfection reagent (Promega, Madison, WI, USA) following the manufacturer’s guidelines. Virus-containing supernatant was collected 48 h post-transfection.

When primary myoepithelial and luminal cells were infected with lentiviral particles, particles were treated with 20 mU/ml neuraminidase (Sigma-Aldrich) at 37 °C for 30 minutes prior to their addition to cells. Particles provided with SMARTchoice promoter selection plates (GE Dharmacon, Lafayette, CO, USA) were also treated with 20 mU/ml neuraminidase prior to their application to cells; fluorescence intensity values were acquired using a FLUOstar Omega fluorescent plate reader (BMG Labtech, Cary, NC, USA).

### Image analysis

To objectively and systemically quantify the spheroid volume, a customised eight-step image analysis process was developed. Z-sections of DAPI-labelled cells were converted into greyscale and used to build the greyscale distribution profile for the set of images. A threshold indicating whether a pixel in the image relates to a cell is then calculated; using this, z-sections are further processed into binary images that indicate cell presence. The pixels indicating cells are then extracted and translated into a geometrically accurate point cloud using original image resolution values.

The generated point cloud contains the spheroid volume of interest, cells that exist outside the spheroid and potential noise from the data capture. To determine the points that represent the spheroid volume of interest, density-based spatial clustering of applications with noise was employed. This detects clusters of points within the 3D space with the largest cluster in terms of the number of points being the spheroid volume. These points are extracted to form the final point cloud. The alpha-shape algorithm is then applied to form triangulated bodies that represent the cell and spheroid volumes. The algorithm requires selection of the alpha radius to compute the bodies, and this parameter was calculated as a function of the image resolution. Using the triangulated bodies, the cell and spheroid volumes are calculated alongside the resultant cell/body ratio.

Initial image analysis was performed using the Python programming language and the Python Imaging Library, SkLearn, NumPy and Matplotlib libraries. The alpha-shape algorithm was performed using MATLAB software and the Computational Geometry toolbox (MathWorks, Natick, MA, USA).

### Statistical analysis

Change in volume ratio between indicated treatments was compared by analysis of variance followed by Tukey’s post hoc test using Prism 5 software (GraphPad Software, La Jolla, CA, USA).

## Additional file

Additional file 1: Supplementary material. **Figure S1.** Promoter efficacy in primary cultures of myoepithelial and luminal cells. **a** Representative images of GFP expression in myoepithelial and luminal cells 48 h following infection with neuraminidase-treated lentiviral particles driving GFP expression under either human/mouse *CMV*, human/mouse *EF1α*, *CAG*, *PGK* and *UBC* promoters. Scale bar = 20 μm. **b** Mean fluorescence intensity (MFI) values of myoepithelial and luminal cells 48 h post-infection with lentiviral particles as in (**a**). Images and values are representative of cells derived from two donors. **Figure S2.** Spheroids formed in Matrigel cultures express markers of both luminal and myoepithelial cells. Expression of cytokeratin (CK) 8 and P-cadherin in spheroids formed in Matrigel from co-culture of isolated myoepithelial and luminal cells over 21 days. Images are representative of cells derived from at least three donors. Scale bar = 20 μm. **Figure S3.** Objective and systematic calculation of cell and spheroid volumes. Representative workflow of spheroid analysis. Raw DAPI z-sections (**a**) are converted into greyscale images and a greyscale distribution profile (**b**). Greyscale images are then converted to binary images using a calculated threshold to indicate cell presence (**c**). The pixels that indicate cells are then translated into a geometrically accurate point cloud using the known image resolutions (**d**). Further post-processing using density-based spatial clustering of applications with noise (DBSCAN) is performed to identify the main body of cells (**e**). The point cloud representing the main spheroid is then extracted (**f**). The alpha-shape algorithm is applied using thresholds set as a function of the image resolutions to form triangulated bodies that represent the cells and body (**g**). The volumes of these bodies are then calculated alongside the resultant cell/body ratio. (PDF 1342 kb)
