# Supplementary material for: A 3D in vitro model of the human breast duct: a method to unravel myoepithelial-luminal interactions in the progression of breast cancer
Source: Breast Cancer Res. 2017 Apr 21;19:50. doi: 10.1186/s13058-017-0843-4 (PMC5399380; doi:10.1186/s13058-017-0843-4)
Supplement: Supplementary file 1 — Figure S1. Promoter efficacy in primary cultures of myoepithelial and luminal cells. a Representative images of GFP expression in myoepithelial and luminal cells 48 h following infection with neuraminidase-treated lentiviral particles driving GFP expression under either human/mouse CMV, human/mouse EF1α, CAG, PGK and UBC promoters. Scale bar = 20 μm. b Mean fluorescence intensity (MFI) values of myoepithelial and luminal cells 48 h post-infection with lentiviral particles as in (a). Images and values are representative of cells derived from two donors. Figure S2. Spheroids formed in Matrigel cultures express markers of both luminal and myoepithelial cells. Expression of cytokeratin (CK) 8 and P-cadherin in spheroids formed in Matrigel from co-culture of isolated myoepithelial and luminal cells over 21 days. Images are representative of cells derived from at least three donors. Scale bar = 20 μm. Figure S3. Objective and systematic calculation of cell and spheroid volumes. Representative workflow of spheroid analysis. Raw DAPI z-sections (a) are converted into greyscale images and a greyscale distribution profile (b). Greyscale images are then converted to binary images using a calculated threshold to indicate cell presence (c). The pixels that indicate cells are then translated into a geometrically accurate point cloud using the known image resolutions (d). Further post-processing using density-based spatial clustering of applications with noise (DBSCAN) is performed to identify the main body of cells (e). The point cloud representing the main spheroid is then extracted (f). The alpha-shape algorithm is applied using thresholds set as a function of the image resolutions to form triangulated bodies that represent the cells and body (g). The volumes of these bodies are then calculated alongside the resultant cell/body ratio. (PDF 1342 kb) [file 13058_2017_843_MOESM1_ESM.pdf]

Supplementary Figure 1. Promoter efficacy in primary cultures of myoepithelial and luminal cells. A) Representative images of GFP expression in myoepithelial and luminal cells 48 hours following infection with neuraminidase-treated lentiviral particles driving GFP expression under either human/mouse *CMV*, human/mouse *EF1α*, *CAG*, *PGK* and *UBC* promoters. Scale bar = 20 μm. **B)** Mean fluorescence intensity (MFI) values of myoepithelial and luminal cells 48 hours post infection with lentiviral particles as in **A**. Images and values representative of cells derived from two donors.

Supplementary Figure 2. Spheroids formed in Matrigel cultures express markers of both luminal and myoepithelial cells. Expression of Cytokeratin (CK) 8 and P-Cadherin in spheroids formed in Matrigel from co-culture of isolated myoepithelial and luminal cells over 21 days. Images representative of cells derived from at least three donors. Scale bar = 20 μm.

Supplementary Figure 3. Objective and systematic calculation of cell and spheroid volumes. Representative workflow of spheroid analysis. Raw DAPI z-sections (**A**) are converted into grey-scale images and a grey-scale distribution profile (**B**). Grey-scale images are then converted to binary images using a calculated threshold to indicate cell presence (**C**). The pixels that indicate cells are then translated into a geometrically accurate point-cloud using the known image resolutions (**D**). Further post-processing using Density-Based Spatial Clustering of Applications with Noise (DBSCAN) is performed to identify the main body of cells (**E**). The point cloud representing the main spheroid is then extracted (**F**). The Alpha Shape algorithm is applied using thresholds set as a function of the image resolutions to form triangulated bodies that represent the cells and body (**G**). The volumes of these bodies are then calculated alongside the resultant cell/body ratio.

Figure S1

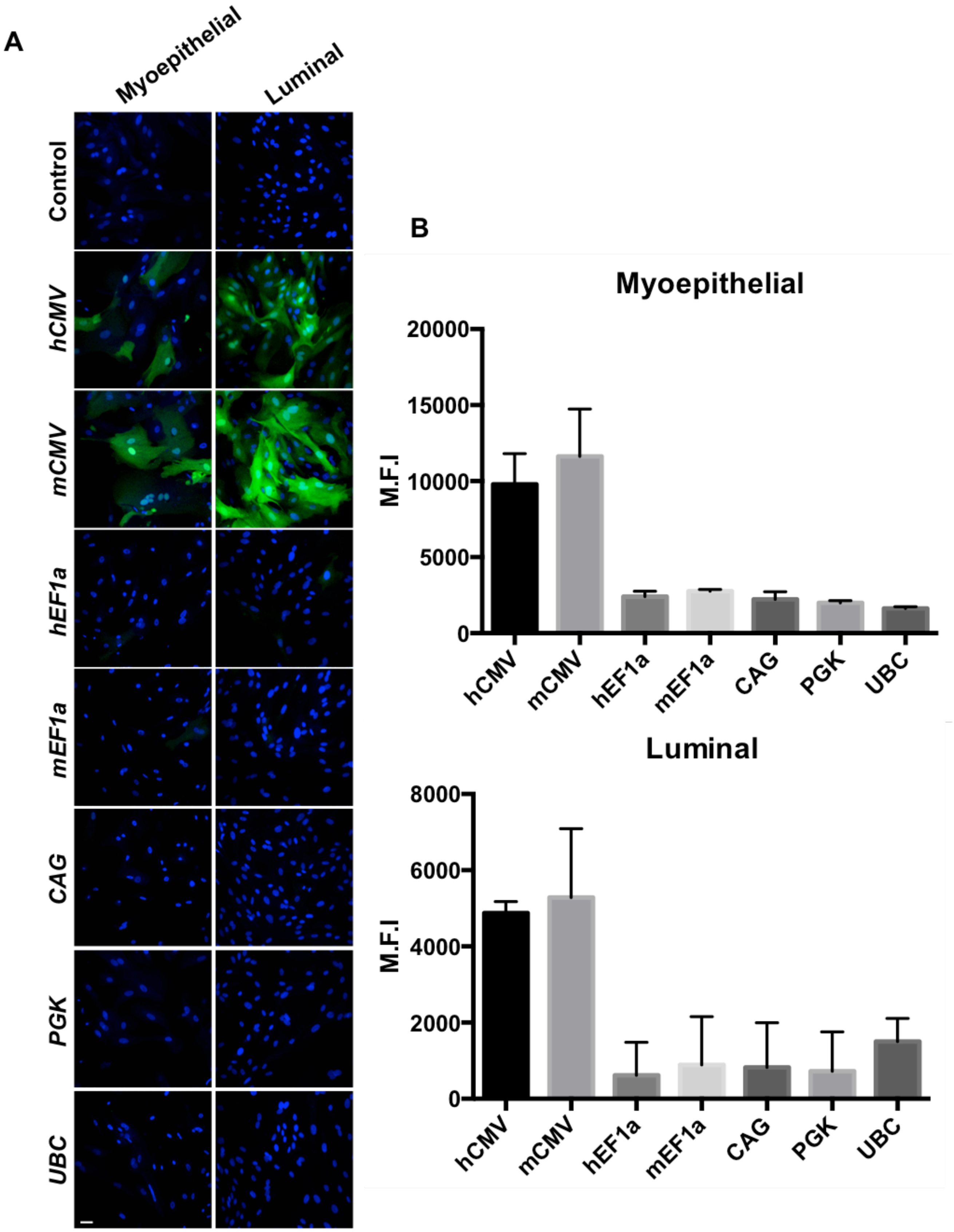
A
Myoepithelial
Luminal
Control
hCMV
mCMV
hEF1a
mEF1a
CAG
PGK
UBC
B
Myoepithelial
20000
15000
10000
5000
0
M.F.I
hCMV
mCMV
hEF1a
mEF1a
CAG
PGK
UBC
Luminal
8000
6000
4000
2000
0
M.F.I
hCMV
mCMV
hEF1a
mEF1a
CAG
PGK
UBC

Figure S2

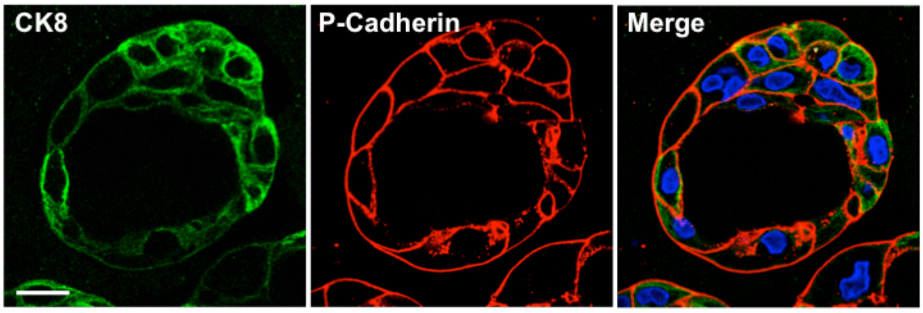

Figure S3

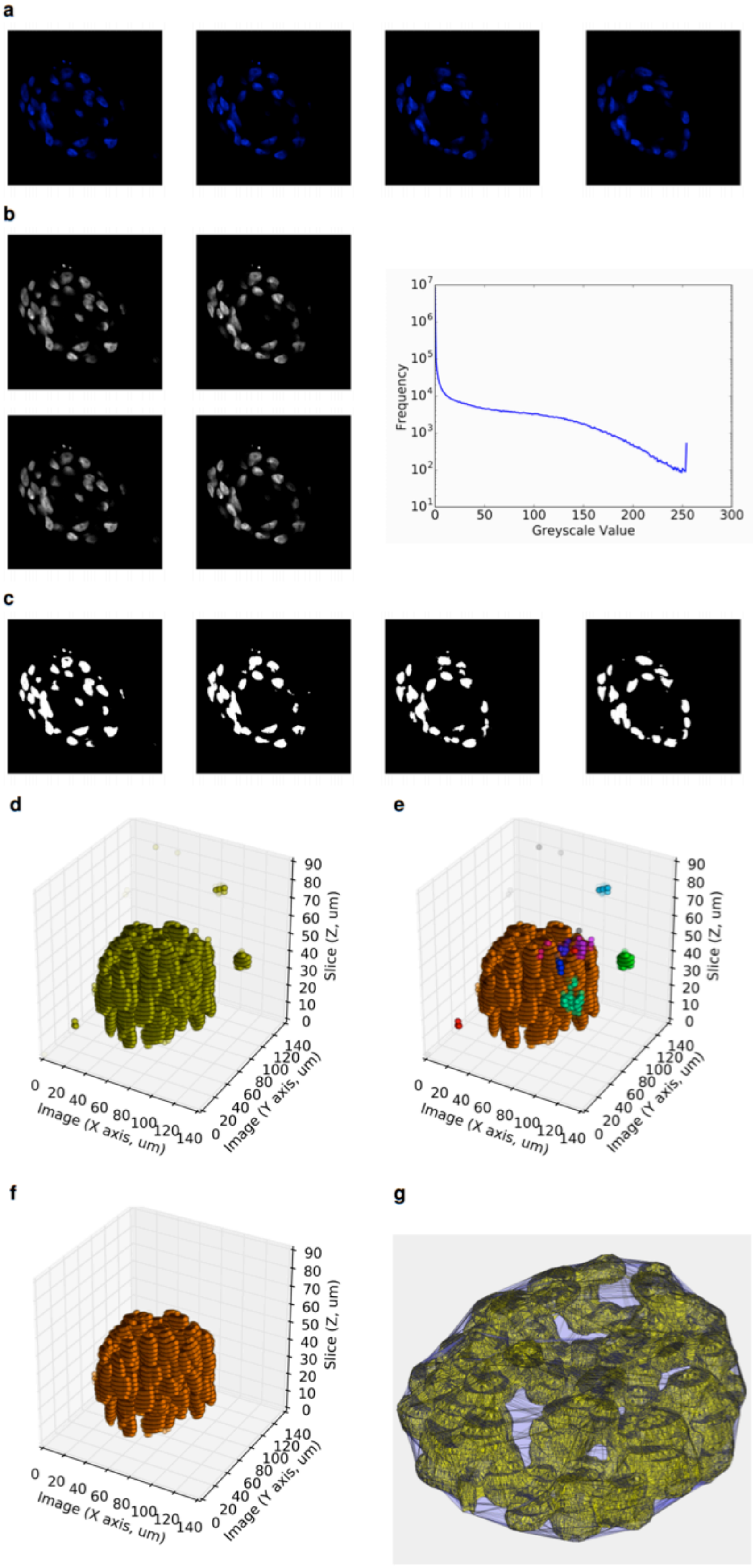
a
b
c
d
e
f
g
Frequency
Greyscale Value
10^1
10^2
10^3
10^4
10^5
10^6
10^7
0 50 100 150 200 250 300
Image (X axis, um)
Image (Y axis, um)
Slice (Z, um)
0 20 40 60 80 100 120 140
0 10 20 30 40 50 60 70 80 90
